# Diagnostic spectrum and clinical outcomes of chronic inflammatory demyelinating polyneuropathy: a multicenter cohort

**DOI:** 10.3389/fneur.2026.1958666

**Published:** 2026-10-01

**Authors:** Reem M. Alhammad, Abdulrahman Ali, Roula Alhadyan, Ahmad R. Abuzinadah, Ahmed K. Bamaga, Muteb Alotaibi, Rawan Albalawi, Raseel Aljthalin, Raed Gasemaltayeb, Talal M. Al-Harbi, Faisal Tarabzouni, Hana Albulaihe, Mohammed H. Alanazy, Meshal M. Alfallaj

**Affiliations:** 1 Department of Internal Medicine, College of Medicine, King Saud University, Riyadh, Saudi Arabia; 2 National Neuroscience Institute, King Fahad Medical City, Riyadh, Saudi Arabia; 3 Department of Neurology, Faculty of Medicine, King Abdulaziz University, Jeddah, Saudi Arabia; 4 Neuromuscular Medicine Unit, King Abdulaziz University Hospital, King Abdulaziz University, Jeddah, Saudi Arabia; 5 Department of Internal Medicine, Neurology Division, International Medicine Center, Jeddah, Saudi Arabia; 6 Department of Pediatrics, Faculty of Medicine, King Abdulaziz University, Jeddah, Saudi Arabia; 7 Prince Sultan Military Medical City, Riyadh, Saudi Arabia; 8 Department of Internal Medicine, Faculty of Medicine, King Abdulaziz University, Rabigh, Saudi Arabia; 9 Department of Neurology, King Fahad Specialist Hospital, Dammam, Saudi Arabia

**Keywords:** chronic inflammatory demyelinating polyneuropathy, EAN/PNS criteria, immune-mediated polyneuropathy, inflammatory polyneuropathy, nerve conduction studies (NCS)

## Abstract

**Background:**

Chronic inflammatory demyelinating polyradiculoneuropathy (CIDP) is the most common immune-mediated demyelinating polyneuropathy, yet data from Arab populations are scarce. We present the first multicenter retrospective Arab cohort describing demographic, clinical, electrophysiological features, and treatment outcomes of CIDP.

**Methods:**

Electronic records from five hospitals in Saudi Arabia were searched between January 2014 and January 2025 for patients diagnosed with CIDP. Patients meeting 2021 EAN/PNS criteria, aged 3 to 80 years, with a minimum follow-up of 3 months were included. Outcome at initial and last visit assessed using MRCSS and INCAT disability scores, with minimal clinically important differences defined as a 4-point and 1-point change in each score, respectively.

**Results:**

Thirty-six (18 male, 18 female) met criteria (33 CIDP,3 possible CIDP). Onset age averaged 33 years (40 years in adult-onset cases). Typical CIDP was predominant (72%), with distal, motor-predominant, and multifocal variants representing 8% each. All motor demyelinating features were upper limb predominant. Maintenance monotherapy, mainly IVIG (42%) or oral steroids (33%), was used to treat 75% of patients. Over 55 months, meaningful improvement was achieved in 89% (INCAT) and 83% (MRCSS) with 3.7- and 11.9-point changes, respectively (*p* < 0.001). Relapses occurred in 42% at a mean of 11 months, mostly due to suboptimal response or non-compliance.

**Conclusion:**

This is the first Arab multicenter CIDP study, which shows earlier disease onset than North American and European cohorts, with a similar clinical phenotype. Despite a 42% relapse rate, most patients showed meaningful improvement. These findings provide a regional benchmark for future prospective studies.

## Introduction

Chronic Inflammatory Demyelinating Polyradiculoneuropathy (CIDP) is an immune-mediated demyelinating disorder of the peripheral nervous system characterized by progressive muscle weakness and/or sensory impairment that progresses over a course of 8 or more weeks (1). Clinical presentations are heterogeneous; typical CIDP is the most common variant, representing half of CIDP cases and characterized by symmetric, proximal and distal, sensory and motor deficits (2). Nerve conduction studies (NCS) typically show slowing of conduction velocity (CV), prolonged distal motor latency (DML), conduction block (CB), temporal dispersion (TD), prolonged distal CMAP duration, abnormal or absent F waves, and abnormal sensory nerve conduction responses (3). Steroids, immunoglobulins, and plasma exchange represent the first-line treatment (4). Two-thirds of CIDP patients have a favorable outcome to first-line treatment with only 10–15% resistant to these treatments (5). Recently, a novel category of disorder termed “autoimmune nodopathies” has been defined by the presence of antibodies (NF155, NF186, CASPR and CNTN1) targeting cell adhesion molecules located at the node/paranode of Ranvier. These disorders share clinical and pathophysiological features with typical CIDP; however, they are regarded as distinct conditions because they require different treatments and are studied separately in clinical research (6, 7). Epidemiological data on CIDP in Saudi Arabia that elucidate clinical features and treatment outcomes are sparse. Herein, we present the first retrospective multicenter cohort reported in our region that depicts the demographic features, clinical manifestations, and electrophysiological findings of CIDP.

## Methods

### Study design and population

Electronic medical records in five tertiary hospitals in Saudi Arabia were reviewed to search for patients diagnosed with CIDP between January 2014 and January 2025. Laboratory values, cerebrospinal fluid analysis, imaging studies, nerve biopsy findings, and neurophysiologic studies were collected. Inclusion criteria were: 1) Patients with CIDP or possible CIDP based on EAN/PNS 2021 diagnostic criteria (3), 2) Age 3 to 80 years at the time of diagnosis, 3) Documentation of treatment regimens used, and 4) A minimum follow-up period of 3 months. Exclusion criteria were: 1) Patients with other causes of neuropathy, such as toxic, neoplastic, metabolic, infectious, or hereditary neuropathy, 2) Patients with nodopathies/paranodopathies. For nerve conduction studies in adult patients, we used age-stratified normative reference data derived from a healthy Saudi Arabian cohort (8, 9). For pediatric patients, age-stratified normative values reported in a North American cohort were used (10). This study was approved by the Ethics Committee of College of Medicine, King Saud University, Riyadh, Saudi Arabia.

### Outcome measures

We assessed clinical outcomes by measuring MRCSS and INCAT scores documented at the patient’s initial and last clinical assessment. Clinically meaningful changes in both MRCSS and INCAT scores (clinical improvement or worsening) were defined as the attainment of minimal clinically important differences (MCIDs) used in large CIDP clinical trials: a 1-point change in the INCAT score or a 4-point change in the MRCSS (11, 12). Relapse was defined as clinical worsening after a documented period of improvement based on the treating physician’s assessment.

### Statistical analysis

Baseline demographic, clinical, electrophysiological, and treatment variables were summarized using frequencies and percentages for categorical data and mean ± standard deviation for continuous variables. Normality of continuous variables was assessed using the Shapiro–Wilk test. Linear regression was used to assess continuous outcome measures (INCAT at last follow-up, change in INCAT, and change in MRCSS). Logistic regression models were used to assess covariates associated with relapse occurrence. Cox proportional hazards regression was used to assess time to relapse. Due to the small sample size (*N* = 36), a parsimonious modeling approach was adopted. Variable selection was based on univariable screening (*p* < 0.20) and clinical relevance, with a limited number of predictors included to avoid overfitting. *p* < 0.05 was considered to indicate statistically significant differences. R-Studio and SPSS were used for statistical analyses.

## Results

A total of 81 patients with demyelinating features on NCS were identified from review of electronic medical charts in five tertiary hospitals in Saudi Arabia. Of these 81 patients, only 36 (18 males and 18 females) met the diagnostic criteria for CIDP. The remaining 45 patients were excluded as they either had incomplete data (*n* = 28), an alternative diagnosis (*n* = 9), or did not fulfill the criteria (*n* = 8) (Figure 1). The mean age at first onset of symptoms and age at confirmed diagnosis was 33 years for all patients and 40 years after excluding pediatric-onset patients (eight patients with onset <18 years of age). Demographic data and clinical characteristics are summarized in Table 1.

**Figure 1:**
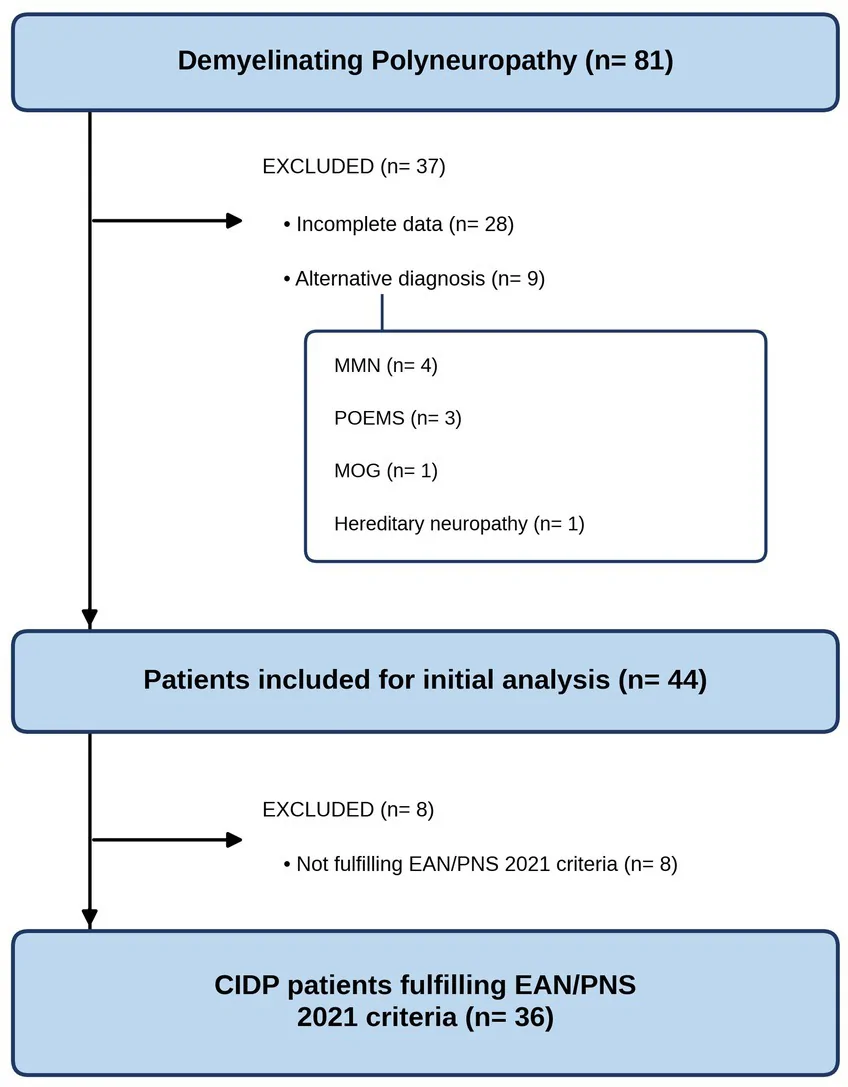
Flow diagram of all patients.

**Table 1:** Demographic data and clinical characteristics in our CIDP cohort.

General features	*N* = 36
Age at symptom onset, years, mean (SD, range)	33 (18, 6–65)
Age of first presentation, years, mean (SD, range)	33 (18, 6–65)
Age at CIDP diagnosis, years, mean (SD, range)	33 (18, 7–65)
Age group, n (%)	
18–30 years	9 (25)
30–50 years	12 (33)
>50 years	7 (19)
Pediatric-onset CIDP, n (%)^§^	8 (22)
Characteristics	*n* (%)
Gender	
Male	18 (50)
Female	18 (50)
Saudi ethnicity	36 (100)
Type 2 diabetes mellitus	8 (22.2)
Hypertension	4 (11)
Smokers	4 (11)
Autoimmune disorders	n (%)
Type 1 diabetes mellitus	1 (3)
SLE	1 (3)
Crohn’s disease	1 (3)
Behcet disease	1 (3)
Multiple sclerosis	1 (3)
Immune thrombocytopenia	1 (3)
Clinical symptoms	*n* (%)
Site of weakness reported by patients	
Upper and lower limb	28 (78)
Lower extremity	4 (11)
Multifocal	3 (8.3)
Dysphagia at presentation	2 (5.6)
Exertional dyspnea	5 (13.9)
Motor examination findings	*n* (%)
Facial weakness on examination	4 (11)
Ptosis on examination	4 (11)
Neck extension weakness^Ỵ^	4 (11)
Neck flexion weakness^Ỵ^	4 (11)
Pattern of weakness	*n* (%)
Symmetric	33 (92)
Asymmetric	3 (8.3)
Distribution of weakness on physical examination	*n* (%)
Distal and proximal upper and lower limbs	23 (64)
Distal upper and lower limbs	4 (11)
Distal lower limbs	4 (11)
Multifocal	3 (8)
Proximal upper and lower limbs	1 (3)
Reflexes (depressed or absent)	*n*/total tested (%)
Triceps	32/35 (91)
Biceps	32/35 (91)
Brachioradialis	32/35 (91)
Knee	33/35 (92)
Ankle	36 (100)

^§^Pediatric-onset CIDP <18 years. ^Ỵ^(Medical Research Council [MRC] = <4).

The most common pattern of muscle weakness was distal and proximal weakness of upper and lower limbs seen in 23 patients (64%), other patterns of predominant muscle weakness are summarized in Table 1. Ankle reflexes were absent or depressed in all patients (100%); other deep tendon reflexes in the upper and lower extremities were absent in 91% of 35 patients (one patient had documentation of ankle reflexes only). Impairments in proprioception and pain sensation were detected in the distal lower extremities (one-third of patients) or in the distal upper and lower extremities (one-third of patients). Vibration sensation was impaired in the distal lower limbs in one-third of patients (Supplementary Table 1).

Nerve conduction studies revealed sural sparing in 13 out of 35 patients tested (37%). Conduction block affecting the median and ulnar nerves in the forearm was detected in 63 and 55%, respectively. All other demyelinating changes (temporal dispersion, slowing of CV, F-wave prolongation, DML prolongation) were noted more frequently in median and ulnar nerves as compared to peroneal and tibial nerves (Table 2). EMG was performed in 24 patients (66%).

**Table 2:** Electrophysiological findings in our CIDP cohort.

Electrodiagnostic feature	*n*/total tested (%)
Sural sparing pattern^¶^	13/35 (37)
Abnormal median SNAP amplitude with normal sural nerve SNAP amplitude	11/35 (31)
Typical CIDP	9/11 (82)
Distal CIDP	2/11 (18)
Abnormal radial SNAP amplitude with normal sural nerve SNAP amplitude †	1/16 (6)
Abnormal median and radial SNAP amplitude with normal sural nerve SNAP amplitude ^†^	1/16 (6)
Conduction block^‡^	
Median nerve conduction block (below elbow to wrist), n (%)	19/30 (63)
Median nerve conduction block (above elbow to axilla), n (%)	3/9 (33)
Ulnar nerve conduction block (below elbow to wrist)	20/36 (56)
Ulnar nerve conduction block (above elbow to axilla)	3/22 (14)
Peroneal nerve conduction block (below fibular head)	4/34 (12)
Temporal dispersion^§^	
Median nerve temporal dispersion	17/32 (53)
Ulnar nerve temporal dispersion	11/30 (37)
Peroneal nerve temporal dispersion	8/32 (25)
Tibial nerve temporal dispersion	5/31 (16)
Distal motor latency prolongation^††^	
Median nerve distal motor latency prolongation	9/36 (25)
Ulnar nerve distal motor latency prolongation	16/36 (44)
Peroneal nerve distal motor latency prolongation	4/36 (11)
Tibial nerve distal motor latency prolongation	6/36 (17)
Reduced conduction velocity^¥^	
Median nerve motor conduction velocity slowing	22/36 (61)
Ulnar nerve motor conduction velocity slowing	21/36 (58)
Peroneal nerve motor conduction velocity slowing	12/36 (33)
Tibial nerve motor conduction velocity slowing	9/36 (25)
Electromyography	24/36 (66)
Fibrillation potentials or positive sharp waves	17/24 (71)
Chronic neurogenic changes^*^	20/24 (83)
Distribution of chronic neurogenic changes	
Proximal upper and lower limbs	1 (5)
Proximal upper limbs	1 (5)
Distal lower limbs	4 (20)
Distal upper and lower limbs	3 (15)
Distal and proximal upper and lower limbs	11 (55)

SNAP, sural nerve action potential. ^¶^Sural sparing: abnormal median or radial SNAP amplitude with normal sural nerve SNAP amplitude (3). ^†^Typical CIDP in each patient. ^‡^ ≥ 30% reduction of the proximal relative to distal negative peak CMAP amplitude, and distal negative peak compound muscle action potential (CMAP) amplitude ≥20% of lower limit of normal (LLN). ^§^ > 30% duration increase between the proximal and distal negative peak CMAP amplitudes. ^††^Motor distal latency prolongation ≥50% above upper limit of normal (ULN). ^¥^Reduction of motor conduction velocity ≥30% below (LLN). *High motor unit potential amplitude and/or duration.

Features of denervation (fibrillation potentials and/or positive sharp waves) were detected in 71% of patients, while chronic neurogenic changes (long duration and/or high-amplitude MUAPs) were present in 83% of patients (Table 2).

Based on EAN/PNS 2021 CIDP criteria, 33 patients (92%) met criteria for CIDP, and 3 patients (8%) met criteria for possible CIDP. Typical CIDP was the most common pattern, seen in 26 patients (72%), followed by the distal variant, motor-predominant variant, and multifocal variant, each found in 3 patients (8%). A single patient had pure motor CIDP (Figure 2). Slowing of conduction velocity, conduction block, and temporal dispersion were the most commonly fulfilled motor criteria, seen in 56, 64, and 50% of patients, respectively. Fulfillment of diagnostic criteria in our cohort is summarized in Table 3. CSF analysis was performed in 32 patients. CSF protein was elevated in 88% of cases, with normal CSF cell count in 90% of patients (Supplementary Table 2). MR imaging of the cervical spine (16 patients), lumbosacral plexus (16 patients), and lumbosacral spine (25 patients) revealed thickening with enhancement of nerve roots/peripheral nerves in 25, 25, and 24% of patients imaged, respectively (Supplementary Table 2). At least one of the nodal/paranodal antibodies (NF155, NF186, CNTN) was tested in 9 of 36 patients, and all were negative (Supplementary Table 3).

**Figure 2:**
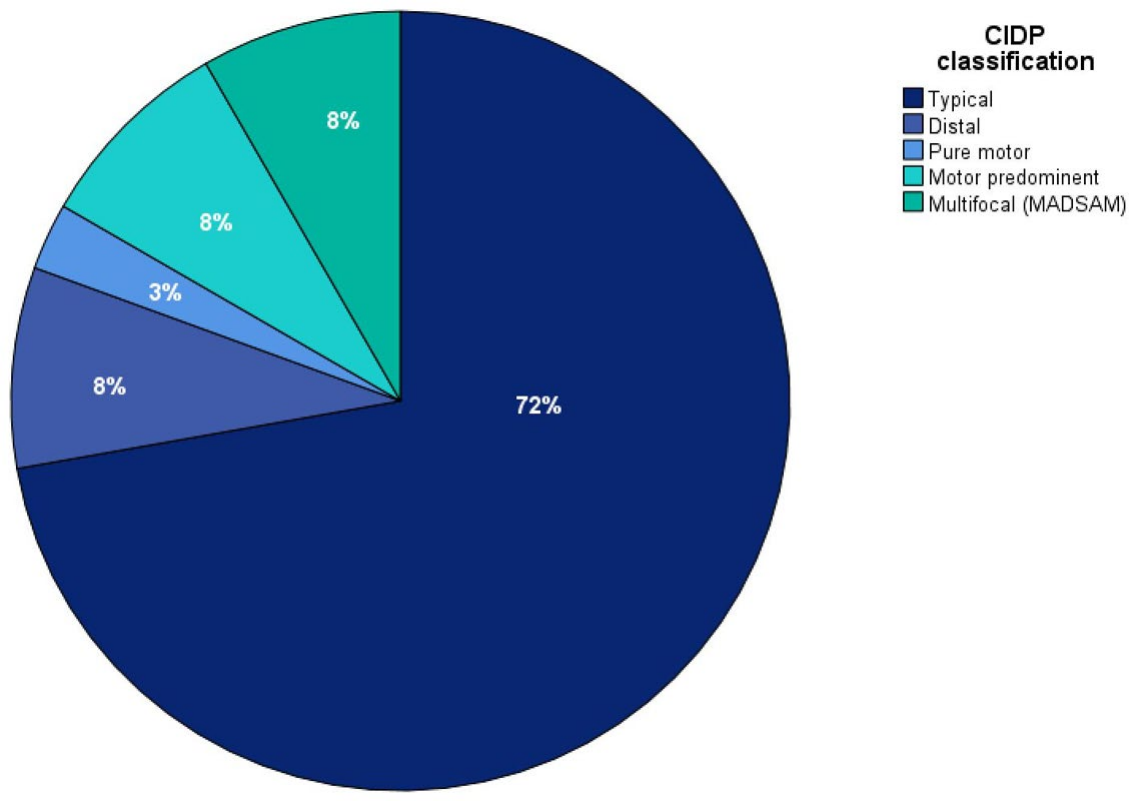
CIDP classification in our cohort.

**Table 3:** CIDP classification and fulfillment of diagnostic criteria in our CIDP cohort.

CIDP classification	*n* (%)
Typical	26 (72)
Distal	3 (8)
Pure motor	1 (3)
Motor predominant	3 (8)
Multifocal (MADSAM)	3 (8)
CIDP diagnostic certainty	*n* (%)
Definite	33 (92)
Possible	3 (8)
Individual criteria	*n*/total tested (%)
Sensory nerve conduction criteria in 2 nerves.	35/36 (97)
Prolonged distal motor latency of >50% above ULN in 2 nerves criteria Ỵ	
Yes	10/36 (28)
Only 1 nerve	9/36 (25)
Slow conduction velocity >30% below LLN in 2 nerves criteria	
Yes	20/36 (56)
Only 1 nerve	9/36 (25)
Prolonged F-wave latency >20% above ULN in 2 nerves or >50% if distal CMAP <80% of LLN criteria.	
Yes	4/32 (13)
Only 1 nerve	7/32 (22)
Absence of F-wave in 2 nerves (If distal CMAP>20% if LLN) + Other demyelinating parameter in > = 1 nerve criteria.	
Yes	4/32 (12.5)
Only 1 nerve	5/32 (16)
Motor conduction block >30% (excluding tibial nerve) and distal CMAP > = 20% of LLL in 2 nerves or in 1 nerve + other demyelinating features in = > 1 nerve except absence of F wave in >1 = nerve criteria	
Yes	23/36 (64)
Only 1 nerve	5/36 (14)
Abnormal temporal dispersion >30% (At least 100% in tibial nerve) > =2 nerve criteria.	
Yes	16/32 (50)
Only 1 nerve	5/32 (16)

^Ỵ^Excluding median neuropathy at the wrist from carpal tunnel syndrome. CMAP, compound muscle action potential; LLN, lower limit of normal; MADSAM, multifocal acquired demyelinating sensory and motor neuropathy; ULN, upper limit of normal.

Variable maintenance therapies were utilized to achieve disease remission in our cohort. Monotherapy was used in 27 patients (75%); among these, 42% received IVIG, and 33% received oral corticosteroids. Combination immunotherapy was used in 9 patients (25%) and includes multiple regimens (Table 4). Only 7 patients (19.4%) were off treatment; reasons for being off therapy include clinical improvement in four patients, pregnancy in two patients, and patient preference in one.

**Table 4:** Immunotherapies used in our CIDP cohort.

Treatment	*n* (%)
Immunotherapy	
Oral steroid	12 (33)
IV immunoglobulins	15 (42)
Azathioprine	6 (17)
Mycophenolate	5 (14)
Plasma exchange	1 (3)
Rituximab	6 (17)
Methotrexate	1 (3)
Frequency of IVIG	10 (63)
Did not receive IVIG	9 (25)
Monthly	13 (36)
q2Weeks	1 (3)
q3Weeks	1 (3)
q6Weeks	6 (17)
q8Weeks	3 (8)
Received only once	3 (9)
Patients on combined treatment	
Monotherapy	27 (75)
2 medications	7 (19)
3 medications	2 (6)
Combined treatment options	*N* = 9
Oral steroid + IVIG	2 (22)
Oral steroid + Azathioprine	1 (11)
IVIG + Azathioprine	2 (22)
IVIG + Rituximab	2(22)
Oral steroid + Rituximab	1 (11)
IVIG + Mycophenolate	1 (11)
IVIG + Mycophenolate + Rituximab	1 (11)
Oral steroid + Mycophenolate + Rituximab	1 (11)
Patients off treatment	7 (19)

IV, intravenous; IVIg, intravenous immunoglobulin.

The mean interval from initial assessment to last documented visit was 55 months. At baseline, the mean INCAT score and MRCSS were 6 (range 1–10) and 42 (range 24–58), respectively. Significant improvements (*p* < 0.001) in INCAT score and MRCSS (defined as attaining MCIDs in either score) were detected for 33 patients (89% based on INCAT and 83% based on MRCSS), at last documented visits compared to initial assessment. The mean changes in INCAT and MRCSS were 3.69 (range: −6 to 9) and 11.94 (range: −6 to 36), respectively, indicating variability in magnitudes of treatment responses among patients (Table 5). A total of 29 patients (81%) showed clinical improvement on both outcome measures (INCAT and MRCSS). Relapses occurred in 15 patients (42%) while on immunotherapy, with a mean interval of 11 months following initiation of immunotherapy. The most reported reasons of relapse documented by treating physicians were treatment failure (47%) and medication non-compliance (40%).

**Table 5:** Baseline functional scores and clinical outcomes in our CIDP cohort.

Outcome	Results
Time from diagnosis to last visit in months	
Mean (SD)	55 (59)
Median (range)	34 (2–299)
Baseline functional scores (prior to initiation of immunotherapies)	
INCAT score (0–10), mean (SD, range)	6 (2.5, 1–10)
MRC sum score (score 0–60), mean (SD, range)	42 (9, 24–58)
Change in functional scores from baseline to last visit	
Δ Change in INCAT, mean (SD)	3.69 (3.4)
Δ Change in MRC, mean (SD)	11.94 (10)
Functional scores at the last visit	
INCAT score at last follow-up (score 0–10), mean (SD)	2.19 (2.7)
MRC sum score at last visit (score 0–60), mean (SD)	53.92 (9)
Change in functional scores following immunotherapies	
INCAT score, *n* (%)	
No change*	3 (8)
Improved**	32 (89)
Worse***	1 (3)
MRC sum score, *n* (%)	
No change*	5 (14)
Improved**	30 (83)
Worse***	1 (3)
Clinical improvement based on a single functional score, *n* (%)	
INCAT score only	3 (75)
MRC sum score only	1 (25)
Clinical improvement based on both INCAT and MRC sum score, *n* (%)	29 (81)
Clinical improvement based on either INCAT or MRC sum score, *n* (%)	33 (92)
No clinical change on INCAT or MRC sum score, *n* (%)	3 (8)
Clinical relapse	
Number of patients who relapsed, *n* (%)	15 (42)
Time to relapse (months after immunotherapy initiation), mean (SD)	11.00 (9)
Reason for relapse, *n*/total relapses (%)	
Treatment failure^Ỵ^	7/15 (47)
Non-compliance	6/15 (40)
Discontinuation of treatment by patient	1 (7)
Not documented	1 (7)

*Same scores from baseline to last visit. **improved by at least 1 (INCAT) or 4 (MRC sum score) points. ***worsened by at least 1 (INCAT) or 4 (MRC sum score) points. ^Ỵ^Worsening of MRC or INACT scores despite being on optimal doses of immunotherapy treatment.

Univariable logistic regression analysis was performed to screen potential predictors of clinical relapse. Variables with *p* < 0.20 were considered for inclusion in a multivariable model. Based on this criterion, prolongation of distal motor latency of the peroneal nerve (*p* = 0.184) was selected. Age (*p* = 0.0608) and weakness distribution (*p* = 0.107) were also retained due to clinical relevance. The multivariable logistic regression model identified no statistically significant predictors of relapse. Multivariable linear regression analysis demonstrated the limited ability of age and gender to predict clinical outcomes. Female gender was significantly associated with higher INCAT scores at last follow-up (3.1) compared to male gender (1.3), with a mean difference of 1.8 (*p* = 0.042), indicating worse functional status, but no significant predictors were identified for change in INCAT or MRCSS.

### Discussion

To our knowledge, this is the first multicenter study describing the clinical, electrophysiological, and treatment outcomes of CIDP in Saudi Arabia. Our study provides insight into potential differences in the clinical characteristics and treatment outcomes in CIDP in an Arab cohort compared to North American or European cohorts.

Onset of CIDP symptoms occurred at a younger age in our patients (mean of 33 years, mean of 40 years after excluding pediatric-onset CIDP) when compared to most European and North American CIDP studies that report symptom onset in the fifth to seventh decades (13, 14). This is likely explained by the relative distribution of age groups in the Saudi population, which is composed mostly of younger adults, with only 3.8% of Saudi citizens aged 65 or older (15). We also observed an equal sex distribution (50% each), in contrast to the male predominance reported in previous studies (13, 16). Clinically, a symmetric pattern of weakness was present in 92% of patients, with combined proximal and distal weakness of all extremities present in 64%. The distribution of CIDP subtypes in our cases aligns closely with published literature; typical CIDP accounted for 72% of cases, with distal, motor-predominant, and multifocal (MADSAM) variants each representing roughly 8%, and only a single patient presented with a pure motor variant. This pattern is broadly consistent with an Italian CIDP database, in which typical CIDP was the most common pattern, while other patterns were less frequent (17). Furthermore, in a CIDP cohort from the United Kingdom, typical CIDP comprised 79% of patients, and the largest component of “CIDP variant” was focal/multifocal forms, accounting for 14% (16).

Applying the 2021 EAN/PNS diagnostic criteria in our patients resulted in 92% meeting criteria for CIDP and 8% fulfilling criteria of possible CIDP. This diagnostic yield lies at the upper end of the range seen in validation cohorts which report sensitivities of approximately 83% for “CIDP” and 93% for “CIDP or possible CIDP” (16). Demyelinating features in NCS were more frequent in upper-extremity nerves as compared to lower-extremity nerves, this is likely explained by earlier onset axonal loss in lower extremities that impede detection of demyelinating features (18). Conduction block was the most frequent neurophysiologic criterion met in our patients and was detected 63% of median motor nerve studies and 56% of ulnar motor nerve studies in the forearm segment. Sural sparing pattern, as defined as an abnormal median or radial SNAP with a preserved sural response, was present in 31 and 6% of our patients, respectively. In a large CIDP subtype-stratified series (138 patients), a sural sparing pattern on NCS was detected in 43% (abnormal median with normal sural) and in 26% (abnormal radial with normal sural) of patients with typical CIDP (19).

Maintenance therapy consisted of monotherapy in 75% of patients. Intravenous immunoglobulin (IVIG) and oral corticosteroids were the two most common first-line agents (42 and 33%, respectively). Combination immunotherapy was reserved for 25% of patients and consisted of a wide range of regimens, mostly including IVIG or corticosteroids with a steroid-sparing immunosuppressant. This is similar to international practice where IVIG and corticosteroids are the most frequent first-line therapies (3).

Outcomes were favorable overall. Clinical improvement was achieved in 89 and 83% of our patients using either INCAT or MRCSS, respectively, while 81% improved applying both scores (Table 5 and Figure 3). These outcomes are similar to those reported by large IVIG trials reporting 70 to 78% of patients improving on at least one outcome score (20, 21). We did not detect a significant correlation between baseline motor compound muscle action potential amplitudes and clinical outcomes. This is contrary to findings of prior studies showing strong correlations between NCS axonal damage and disability in both first diagnosis and advanced disease stages (22, 23). Relapses occurred in 42% of patients receiving immunotherapy, with a mean of 11 months after treatment initiation. “Treatment failure” and “non-compliance” together accounted for the great majority of relapses. The relapse rate is consistent with long-term observational data showing that relapse is common in CIDP patients whose maintenance immunotherapy is tapered despite patient adherence (24). Evaluating predictors of relapse and treatment outcomes was limited by our small sample size.

**Figure 3:**
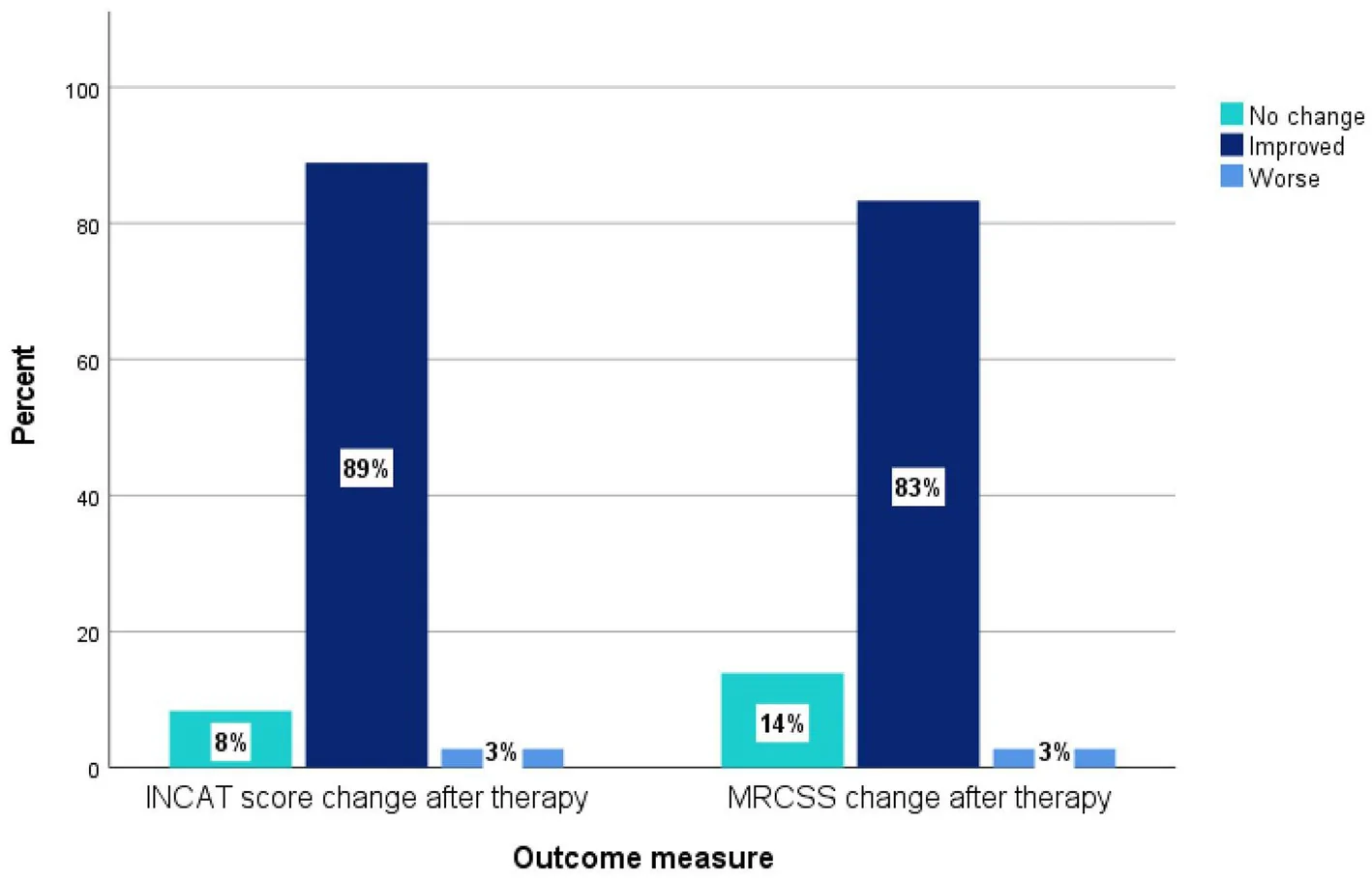
INCAT and MRCSS scores at last documented assessment (Mean 55 months).

## Conclusion

In conclusion, our study reveals a younger age of onset of CIDP in an Arab ethnic cohort as compared to North American and European populations, but it is phenotypically similar, with typical CIDP representing the most frequent type. Demyelinating features on NCS were more frequently detected in upper extremity nerves. Despite the significant relapse rate of 42, 92% of our patients ultimately showed clinical improvement on either INCAT or MRCSS at the last documented assessments. The retrospective design and small sample size limited analysis of predictors of treatment outcomes and correlation with NCS findings. Larger, prospective, population-based studies are needed to more accurately delineate clinical characteristics and treatment outcomes of CIDP in our region.

## Data Availability

The raw data supporting the conclusions of this article will be made available by the authors, without undue reservation.

## References

[1] Kuwabara S. Typical CIDP: update of the pathogenesis, diagnosis, and treatment. Brain Nerve. (2024) 76:515–9. doi: 10.11477/mf.1416202637, 38741490

[2] Mathey EK Park SB Hughes RAC Pollard JD Armati PJ Barnett MH . Chronic inflammatory demyelinating polyradiculoneuropathy: from pathology to phenotype. J Neurol Neurosurg Psychiatry. (2015) 86:973–85. doi: 10.1136/jnnp-2014-309697, 25677463 PMC4552934

[3] Van Den Bergh PYK Van Doorn PA Hadden RDM Avau B Vankrunkelsven P Allen JA . European academy of neurology/peripheral nerve society guideline on diagnosis and treatment of chronic inflammatory demyelinating polyradiculoneuropathy: report of a joint task force—second revision. J Peripher Nerv Syst. (2021) 26:242–68. doi: 10.1111/jns.12455, 34085743

[4] Fehmi J Bellanti R Misbah SA Bhattacharjee A Rinaldi S. Treatment of CIDP. Pract Neurol. (2023) 23:46–53. doi: 10.1136/pn-2021-002991, 36109154

[5] Gogia B. Cabrero F.R. Suheb M.Z.K. Lui F. Rai P.K. Chronic inflammatory demyelinating polyradiculoneuropathy. In: StatPearls [Internet]. StatPearls Publishing; (2024). Available online at: https://www.ncbi.nlm.nih.gov/sites/books/NBK563249/ (Accessed June 29, 2025) 33085396

[6] Cifuentes-Diaz C Dubourg O Irinopoulou T Vigny M Lachkar S Decker L . Nodes of Ranvier and paranodes in chronic acquired neuropathies. PLoS One. (2011) 6:e14533. doi: 10.1371/journal.pone.0014533 PMC3022580 21267074

[7] Stathopoulos P Alexopoulos H Dalakas MC. Autoimmune antigenic targets at the node of Ranvier in demyelinating disorders. Nat Rev Neurol. (2015) 11:143–56. doi: 10.1038/nrneurol.2014.260, 25623793

[8] Alanazy MH Alkhawajah NM Aldraihem MO Muayqil T. Electrodiagnostic reference data for motor nerve conduction studies in Saudi Arabia. NSJ. (2020) 25:25–31. doi: 10.17712/nsj.2020.1.20190067, 31982892 PMC8015621

[9] Alanazy MH Muayqil T Aldraihem MO Alkhawajah NM. Electrodiagnostic reference data for sensory nerve conduction studies in Saudi Arabia. NSJ. (2020) 25:112–22. doi: 10.17712/nsj.2020.2.20190068, 32351248 PMC8015524

[10] Ryan CS Conlee EM Sharma R Sorenson EJ Boon AJ Laughlin RS. Nerve conduction normal values for electrodiagnosis in pediatric patients. Muscle Nerve. (2019) 60:155–60. doi: 10.1002/mus.26499, 31032944

[11] Merkies ISJ Van Nes SI Hanna K Hughes RAC Deng C. Confirming the efficacy of intravenous immunoglobulin in CIDP through minimum clinically important differences: shifting from statistical significance to clinical relevance. J Neurol Neurosurg Psychiatry. (2010) 81:1194–9. doi: 10.1136/jnnp.2009.194324, 20647554

[12] Hughes RA Donofrio P Bril V Dalakas MC Deng C Hanna K . Intravenous immune globulin (10% caprylate-chromatography purified) for the treatment of chronic inflammatory demyelinating polyradiculoneuropathy (ICE study): a randomised placebo-controlled trial. Lancet Neurol. (2008) 7:136–44. doi: 10.1016/S1474-4422(07)70329-0, 18178525

[13] Broers MC Bunschoten C Nieboer D Lingsma HF Jacobs BC. Incidence and prevalence of chronic inflammatory demyelinating Polyradiculoneuropathy: a systematic review and Meta-analysis. Neuroepidemiology. (2019) 52:161–72. doi: 10.1159/000494291, 30669140 PMC6518865

[14] Mahdi-Rogers M Hughes RAC. Epidemiology of chronic inflammatory neuropathies in southeast E ngland. Eur J Neurol. (2014) 21:28–33. doi: 10.1111/ene.12190, 23679015

[15] Population Estimates Publication 2024. Riyadh: General Authority for Statistics, Kingdom of Saudi Arabia (2024). Available online at: doi: https://www.stats.gov.sadocuments/20117/2435273/Population+Estimates+Publication+2024+EN.pdf/7d123c57-1626-7d2f-ba7f-8a719f928f28

[16] Rajabally YA Afzal S Loo LK Goedee H. Application of the 2021 EAN/PNS criteria for chronic inflammatory demyelinating polyneuropathy. J Neurol Neurosurg Psychiatry. (2021) 93:1247–52. doi: 10.1136/jnnp-2022-329633, 36190956

[17] De Lorenzo A Liberatore G Doneddu PE Manganelli F Cocito D Briani C . Impact of 2021 European academy of neurology/peripheral nerve society diagnostic criteria on diagnosis and therapy of chronic inflammatory demyelinating polyradiculoneuropathy variants. Eur J Neurol. (2024) 31:e16190. doi: 10.1111/ene.16190, 38165011 PMC11235819

[18] Rajabally YA Narasimhan M. Distribution, clinical correlates and significance of axonal loss and demyelination in chronic inflammatory demyelinating polyneuropathy. Eur J Neurol. (2011) 18:293–9. doi: 10.1111/j.1468-1331.2010.03138.x, 20602631

[19] Shibuya K Tsuneyama A Misawa S Suzuki Y‐i Suichi T Kojima Y . Different patterns of sensory nerve involvement in chronic inflammatory demyelinating polyneuropathy subtypes. Muscle Nerve. (2022) 66:131–5. doi: 10.1002/mus.27530, 35307862

[20] Merkies ISJ Van Schaik IN Léger J Bril V van Geloven N Hartung H‐P . Efficacy and safety of IVIG in CIDP: combined data of the PRIMA and PATH studies. J Peripher Nerv Syst. (2019) 24:48–55. doi: 10.1111/jns.12302, 30672091 PMC6594229

[21] Bus SRM Broers MC Lucke IM Bunschoten C van Lieverloo GGA Adrichem ME . Clinical outcome of CIDP one year after start of treatment: a prospective cohort study. J Neurol. (2022) 269:945–55. doi: 10.1007/s00415-021-10677-5, 34173873 PMC8782785

[22] Grüter T Motte J Bulut Y Kordes A Athanasopoulos D Fels M . Axonal damage determines clinical disability in chronic inflammatory demyelinating polyradiculoneuropathy (CIDP): a prospective cohort study of different CIDP subtypes and disease stages. Eur J Neurol. (2022) 29:583–92. doi: 10.1111/ene.15156, 34687104

[23] Wannarong T Skolka MP Rattanathamsakul N Swart G Dyck JB Berini SE . CIDP treatment outcomes correlation with first nerve conduction changes: ascertainment of initial and long-term responders. J Peripheral Nervous Sys. (2025) 30:e70017. doi: 10.1111/jns.70017, 40189868

[24] Niu J Zhang L Hu N Cui L Liu M. Long-term follow-up of relapse and remission of CIDP in a Chinese cohort. BMJ Neurol Open. (2024) 6:e000651. doi: 10.1136/bmjno-2024-000651, 38770161 PMC11103238

