# Supplementary material for: Diagnostic spectrum and clinical outcomes of chronic inflammatory demyelinating polyneuropathy: a multicenter cohort
Source: Front Neurol. 2026 Oct 1;17:1958666. doi: 10.3389/fneur.2026.1958666 (PMC13670914; doi:10.3389/fneur.2026.1958666)
Supplement: Supplementary file 1 [file Table_1.DOCX]

**Supplementary tables**

**Supplementary Table 1. Distribution of sensory examination findings in our CIDP cohort.**

| **Sensory examination findings** | **Vibration**  **n/total tested (%)** | **Proprioception**  **n/total tested (%)** | **Pinprick**  **n/total tested (%)** | **Light touch n/total tested (%)** | **Temperature n/total tested (%)** |
| --- | --- | --- | --- | --- | --- |
| **Normal** | 11/30 (31%) | 10/26 (38%) | 9/35 (26%) | 7/20 (35%) | 8/21 (38%) |
| **Reduced in distal lower limbs** | 11/30 (31%) | 8/26 (31%) | 10/35 (29%) | 5/20 (25%) | 7/21 (33%) |
| **Reduced in distal upper limbs** | 0 | 0 | 1/35 (3%) | 0 | 0 |
| **Reduced in distal and proximal upper limbs** | 0 | 0 | 1/35 (3%) | 0 | 0 |
| **Reduced in distal and proximal lower limbs** | 0 | 0 | 1/35 (3%) | 1/20 (5%) | 0 |
| **Reduced in distal upper and lower limbs** | 5/30 (14%) | 8/26 (31%) | 10/35 (29%) | 4/20 (20%) | 4/21 (19%) |
| **Reduced in one focal limb** | 0 | 0 | 1/35 (3%) | 1/20 (5%) | 0 |
| **Reduced in proximal and distal upper and lower extremities** | 3/30 (8%) | 0 | 2/35 (6%) | 2/20 (10%) | /212 (10%) |

**Supplementary Table 2. Cerebrospinal fluid and Magnetic resonance imaging findings in our CIDP cohort.**

| **Cerebrospinal fluid analysis ^¥^** | **n/total tested (%)** |
| --- | --- |
| **Cell count (cells/uL)** |  |
| Normal | 29/32 (90) |
| Elevated | 3/32 (9) |
| **Glucose (mmol/L)** |  |
| Normal | 28/32 (88) |
| Elevated | 4/32 (13) |
| **Protein (g/L)** |  |
| Normal | 4/32 (13) |
| Elevated | 28 (88) |
| **MRI features of cervical roots** | **n/total tested (%)** |
| Normal | 10/16 (63) |
| Only enhancement | 2/16 (13) |
| Both enhancement and thickening | 4/16 (25) |
| **MRI features of brachial plexus** | **n/total tested (%)** |
| Normal | 10/12 (83) |
| Only enhancement | 0 |
| Both enhancement and thickening | 2/12 (17) |
| **MRI features of lumbosacral roots** | **n/total tested (%)** |
| Normal | 12/25 (48) |
| Only enhancement | 3/25 (12) |
| Both enhancement and thickening | 6/25 (24) |
| Only thickening/enlargement | 4/25 (16) |
| **MRI features of lumbosacral plexus** | **n/total tested (%)** |
| Normal | 10/16 (63) |
| Only enhancement | 1/16 (6) |
| Both enhancement and thickening | 4/16 (25) |
| Only thickening/enlargement | 1/16 (6) |
| **MRI features of 3^rd^ & 6^th^ cranial nerves** | **n/total tested (%)** |
| Normal | 14/18 (78) |
| Only enhancement | 0 |
| Both enhancement and thickening | 3/18 (17) |
| Only thickening/enlargement | 1/18 (6) |
| **MRI features of 7^th^ cranial nerve** | **n/total tested (%)** |
| Normal | 14/17 (82) |
| Only enhancement | 1/17 (6) |
| Both enhancement and thickening | 2/17 (12) |

^¥^ Normal reference values for CSF: 1) protein= 0.15-0.45 g/l, 2) glucose= 2.22-3.89 mmol/L, 3) cell count= 0-5/uL

**Supplementary Table 3 . Patients with tested Nodal/paranodal antibodies (N=9)**

| **Antibody** | **Number of positive results /total tests (%)** |
| --- | --- |
| NF155 | 0/7 (0) |
| NF186 | 0/4 (0) |
| CNTN | 0/7 (0) |

NF155, Neurofascin-155; NF186, Neurofascin-155, CNTN: Contactin. No patients were tested for CASPR1.
